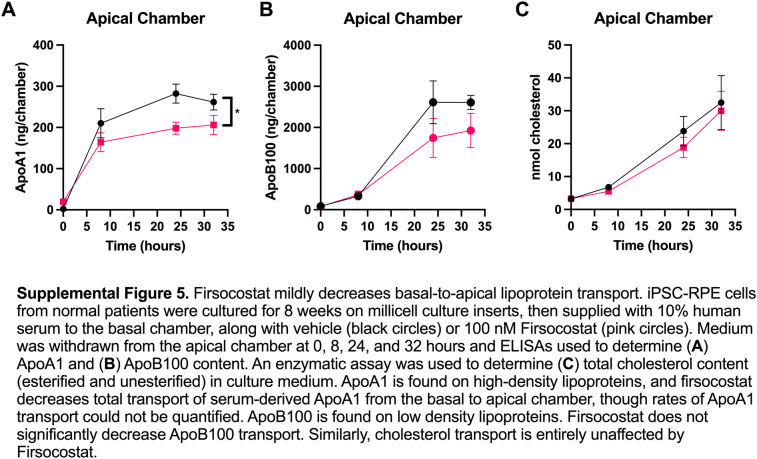# Acetyl-CoA carboxylase inhibition increases retinal pigment epithelial cell fatty acid flux and restricts apolipoprotein efflux

**DOI:** 10.1016/j.jbc.2024.107772

**Published:** 2024-09-12

**Authors:** Daniel T. Hass, Kriti Pandey, Abbi Engel, Noah Horton, Cameron D. Haydinger, Brian M. Robbings, Rayne R. Lim, Martin Sadilek, Qitao Zhang, Gillian A. Gulette, Amy Li, Libin Xu, Jason M.L. Miller, Jennifer R. Chao, James B. Hurley

**Affiliations:** 1 Department of Biochemistry, University of Washington, Seattle, Washington, USA; 2 Center for Developmental Biology and Regenerative Medicine, Seattle Children’s Research Hospital, Seattle, Washington, USA; 3 Department of Laboratory Medicine and Pathology, University of Washington School of Medicine, Seattle, Washington, USA; 4 Department of Ophthalmology, University of Washington, Seattle, Washington, USA; 5 Department of Chemistry, University of Washington, Seattle, Washington, USA; 6 Kellogg Eye Center, University of Michigan, Ann Arbor, Michigan, USA; 7 Department of Medicinal Chemistry, University of Washington, Seattle, Washington, USA

**Keywords:** retinal pigment epithelium, age-related macular degeneration, apolipoprotein E (ApoE), beta-oxidation, retinal degeneration, energy metabolism, fatty acid

## Abstract

Lipid-rich deposits called drusen accumulate under the retinal pigment epithelium (RPE) in the eyes of patients with age-related macular degeneration and Sorsby’s fundus dystrophy (SFD). Drusen may contribute to photoreceptor degeneration in these blinding diseases. Stimulating β-oxidation of fatty acids could decrease the availability of lipid with which RPE cells generate drusen. Inhibitors of acetyl-CoA carboxylase (ACC) stimulate β-oxidation and diminish lipid accumulation in fatty liver disease. In this report, we test the hypothesis that an ACC inhibitor, Firsocostat, can diminish lipid deposition by RPE cells. We probed metabolism and cellular function in mouse RPE-choroid tissue and human RPE cells. We used ^13^C_6_-glucose, ^13^C_16_-palmitate, and gas chromatography–linked mass spectrometry to monitor effects of Firsocostat on glycolytic, Krebs cycle, and fatty acid metabolism. We quantified lipid abundance, apolipoprotein E levels, and vascular endothelial growth factor release using liquid chromatography–mass spectrometry, ELISAs, and immunostaining. RPE barrier function was assessed by trans-epithelial electrical resistance (TEER). Firsocostat-mediated ACC inhibition increases β-oxidation, decreases intracellular lipid levels, diminishes lipoprotein release, and increases TEER. When human serum or outer segments are used to stimulate lipoprotein release, fewer lipoproteins are released in the presence of Firsocostat. In a culture model of SFD, Firsocostat stimulates fatty acid oxidation, increases TEER, and decreases apolipoprotein E release. We conclude that Firsocostat remodels RPE metabolism and can limit lipid deposition. This suggests that ACC inhibition could be an effective strategy for diminishing pathologic drusen in the eyes of patients with age-related macular degeneration or SFD.

Age-related macular degeneration (AMD) is a retinal degenerative disease and the leading cause of blindness in older adults (1). The ultimate cause of degeneration in AMD is unknown, but age, smoking, and circulating HDL-cholesterol are among the risk factors (2). Photoreceptor degeneration in AMD is associated with the accumulation of soft drusen, basal laminar deposits, and subretinal drusenoid deposits (3). These deposits, located between the RPE and Bruch’s membrane, are rich in lipids (4, 5, 6, 7, 8) and lipoproteins (9, 10, 11). Drusen are thought to arise from retinal pigment epithelium (RPE) cells (12). The hydrophobic nature of these lipid-rich structures could impede transport of hydrophilic metabolic fuels like glucose to the RPE and retina (13). A diminished supply of nutrients may then contribute to photoreceptor degeneration in AMD (13). As with AMD, lipid-rich deposits also accumulate with mutations in Timp3 that cause Sorsby’s fundus dystrophy (SFD), but with an earlier onset of symptoms (14, 15).

If a hydrophobic barrier generated by drusenoid structures compromises photoreceptor viability, then slowing its formation or removing it could slow or stop degeneration (16, 17, 18). Our goal is to slow the formation of drusen by stimulating fatty acid oxidation and limit the availability of fatty acids that can contribute to the synthesis of lipids that form drusen.

The family of Acetyl-CoA carboxylases (ACCs) consists of two iso-enzymes that control the balance between lipid synthesis and oxidation. The product of ACCs is malonyl-CoA. Malonyl-CoA produced by cytosolic ACC1 is a substrate for fatty acid synthesis (19, 20). Malonyl-CoA synthesized on the mitochondrial outer membrane by ACC2 prevents oxidation of long-chain fatty acids in mitochondria by inhibiting carnitine palmitoyl transferase (CPT) (21). Esterification of long chain fatty acids to carnitine by CPT is required for long-chain fatty acid import and oxidation in the mitochondrial matrix (22).

ACCs must dimerize to produce malonyl-CoA. Firsocostat (also referred to as GS-0976 or ND-630) is a small molecule that inhibits dimerization of both ACC isoforms (23). It limits malonyl-CoA formation, decreases circulating lipid levels, and decreases hepatic lipid accumulation (23). Firsocostat and other ACC inhibitors are being pursued as a potential therapeutic for nonalcoholic fatty liver disease (23, 24, 25, 26). Our goal is to determine whether inhibition of ACCs using Firsocostat can limit production or accumulation of lipoproteins by RPE.

We investigated the impact of Firsocostat-mediated ACC inhibition on fatty acid oxidation, lipid levels, apolipoprotein transport, and deposition in cultured mouse RPE-choroid and human RPE cells. Firsocostat increases the rate of fatty acid oxidation, remodels lipid composition, and decreases apolipoprotein export by RPE cells. Several different ACC inhibitors have been in clinical trials for the treatment of nonalcoholic fatty liver disease (26, 27, 28). Our data suggest that in addition to treating liver disease, these inhibitors could potentially also limit a pathological increase in drusen in AMD and SFD.

## Results

### Firsocostat increases fatty acid oxidation in mouse RPE-choroid eyecups

**Figure 1:**
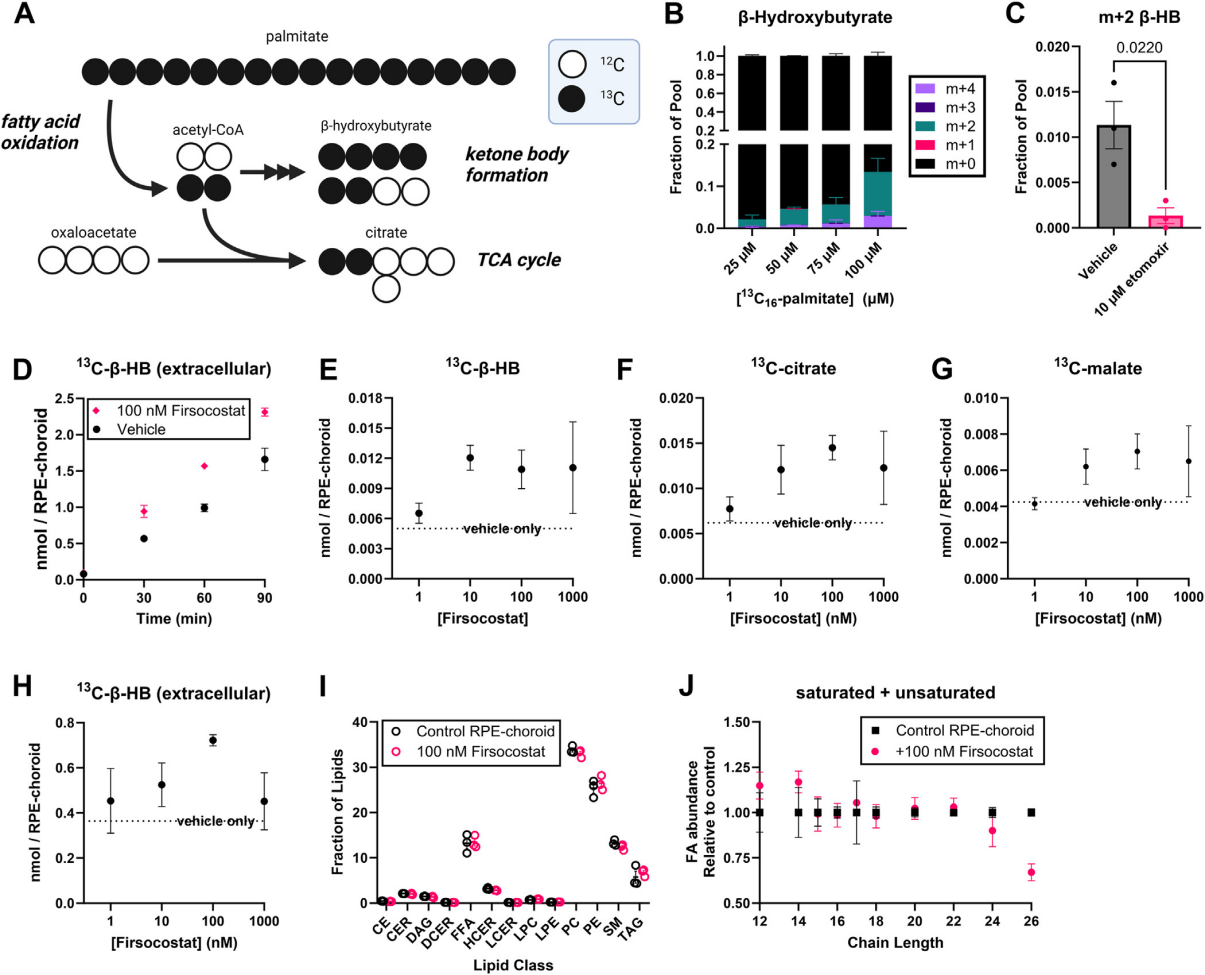
**Firsocostat increases fatty acid oxidation in mouse RPE-choroid tissue.***A*, oxidation of a ^13^C_16_-palmitate molecule in mitochondria produces eight acetyl-CoA molecules, each with two ^13^C atoms (m+2 labeled). Each labeled acetyl-CoA can enter the Krebs cycle to make m+2 citrate or can be used to synthesize ketone bodies such as β-hydroxybutyrate (β-HB). Because ketone bodies are synthesized from two acetyl-CoA molecules, they can be labeled from ^13^C_16_-palmitate as m+2 or m+4. *B*, increasing the extracellular concentration of ^13^C_16_-palmitate for 30 min causes greater m+2 and m+4 labeling of β-hydroxybutyrate (β-HB) (n = 3). *C*, the CPT inhibitor etomoxir suppresses ^13^C accumulation on β-HB over 30 min by (n = 3). *D*, β-HB accumulates ^13^C and is exported to KRB buffer linearly with time (n = 3), an effect augmented by the application of 100 nM Firsocostat. Application of Firsocostat to RPE-choroid for 1 h increases accumulation of ^13^C from palmitate on (*E*) intracellular β-HB, (*F*) intracellular citrate, (*G*) intracellular malate, and (*H*) extracellular β-HB. The effect of Firsocostat is concentration-dependent, with a maximal effect at ∼100 nM (n = 3). *I*, after 6 h, 100 nM Firsocostat does not alter the distribution of lipid classes, though when fatty acid tails are summed across all classes, 100 nM Firsocostat appears to (*J*) decrease the proportion of very long chain fatty acid tails (n = 3). CE, cholesterol esters; CER, ceramides; DAG, diacylglycerols; DCER, dihydroceramides; FFA, free fatty acids; HCER, hexosylceramides; LCER, lactosylceramides; LPC, lysophosphatidylcholines; PC, phosphatidylcholines; PE, phosphatidylethanolamines; SM, sphingomyelins; TAG, triacylglycerols.

We probed fatty acid oxidation (FAO) in this study by tracing the ^13^C label on ^13^C_16_-palmitate. β-oxidation of ^13^C_16_-palmitate (where all carbons of the palmitate molecule carry the ^13^C isotope) produces acetyl-CoA that is two atomic mass units heavier (m+2) than normal acetyl-CoA. Thus, when ^13^C_16_-palmitate–derived m+2 acetyl-CoA is incorporated into citrate (in the Krebs cycle) or combined to make β-hydroxybutyrate (β-HB; a ketone body), each should be m+2 or m+4 labeled, corresponding to incorporation of one or two m+2-labeled acetyl-CoA molecules (Fig. 1*A*).

We determined whether mouse RPE-choroid performs sufficient FAO to detect labeling on downstream metabolic by-products. We provided RPE-choroid with 25 to 100 μM ^13^C_16_-palmitate (conjugated to bovine serum albumin) in Krebs-ringer bicarbonate (KRB) buffer supplemented with 5 mM unlabeled glucose for 30 min. There is a concentration-dependent increase in the labeling of tissue β-HB (Fig. 1*B*). β-HB was predominantly m+0, m+2, or m+4 labeled, supporting the hypothesis that the ^13^C is derived from β-oxidation of ^13^C_16_-palmitate to ^13^C-acetyl-CoA. Oxidation of long-chain fatty acids such as palmitate requires carnitine acyltransferases, which are inhibited by etomoxir (29). Etomoxir diminishes the accumulation of ^13^C from 50 μM ^13^C_16_-palmitate in RPE-choroid tissue over 30 min (Fig. 1*C*). The generation of ^13^C-β-HB and export into medium is linear over time (Fig. 1*D*). These data show that we can assess the oxidation of long-chain free fatty acids by mouse RPE-choroid.

Malonyl-CoA produced by ACCs inhibits carnitine acylation to long-chain free fatty acids and thus their import into mitochondria for β-oxidation (21). ACCs can be inhibited by the small molecule Firsocostat. By blocking the production of malonyl-CoA, Firsocostat dis-inhibits FAO in hepatocytes (23). We determined whether Firsocostat produces the same effect in mouse RPE-choroid. Firsocostat (100 nM) increases the generation and export of ^13^C- β-HB from 50 μM ^13^C_16_-palmitate (Fig. 1*D*), confirming its ability to induce FAO in the RPE. Next, we determined an optimal concentration of Firsocostat to test. We incubated freshly dissected mouse RPE-choroid tissue in 50 μM ^13^C_16_-palmitate, 5 mM unlabeled glucose, and 0, 1, 10, 100, or 1000 nM Firsocostat for 60 min. Firsocostat increased ^13^C labeling of tissue β-HB (Fig. 1*E*), citrate (Fig. 1*F*), and malate (Fig. 1*G*). ^13^C labeled β-HB was also found in culture medium (Fig. 1*H*), suggesting it is both produced and exported. Fractional labeling of β-HB in tissue and culture medium are similar, but labeling is more dilute in medium. Notably, retinas are also able to oxidize ^13^C_16_-palmitate to make β-HB and citrate, though unlike RPE-choroid, their ability to do so is not enhanced by Firsocostat (Fig. S2, *A* and *B*). OATP1B1 and OATP1B3 are the main Firsocostat transporters in the liver. We used a web-based tool to determine levels of OATP1B1 and OATP1B3 transcript in the adult human retina and RPE (integrated across multiple datasets; https://eyeintegration.nei.nih.gov/). OATP1B1 and both transcript variants of OATP1B3 are more highly expressed in the RPE than the retina (Fig. S2, *C*–*E*). This may explain the limited effectiveness of Firsocostat accelerating FAO in the retina; it may not be transported into retina tissue.

We determined whether lipid profiles in RPE-choroid also are affected by Firsocostat. We exposed freshly dissected mouse RPE-choroid to 50 μM unlabeled palmitate, 5 mM glucose, and vehicle or 100 nM Firsocostat in KRB for 6 h. Tissue was flash-frozen, and lipid profiles assessed on the Lipidyzer mass spectrometry platform. Firsocostat did not appear to alter the abundance of any lipid class (Fig. 1*I*) but does affect the distribution of fatty acid tails on lipids, with a decrease in very long-chain fatty acid tails (C26) and a non-statistically significant increase in species carrying short chain fatty acid tails (Fig. 1*J*). These data were initially unexpected because an increase in mitochondrial FAO should selectively affect fatty acids with chain lengths ≤20 carbons. Generally, fatty acids with more than 20 carbons are oxidized by peroxisomes. Malonyl-CoA is an essential substrate for the elongation of very long chain fatty acid enzymes, so reduction in malonyl-CoA levels with Firsocostat may also affect synthesis of very long chain fatty acids (30). Additionally, malonyl-CoA inhibits peroxisomal carnitine acyltransferases (31), so the reduction in malonyl-CoA levels by Firsocostat may promote peroxisomal oxidation of fatty acids with chain lengths >22 carbons. However, all of these changes were relatively low in magnitude, and the time required for changes in fatty acid oxidation to affect fatty acid composition may be sufficiently long that tissues require a longer incubation with Firsocostat for changes in FAO to translate to an altered lipid profile (32).

### ACC inhibition stimulates β-oxidation of fatty acids by cultured human RPE cells

**Figure 2:**
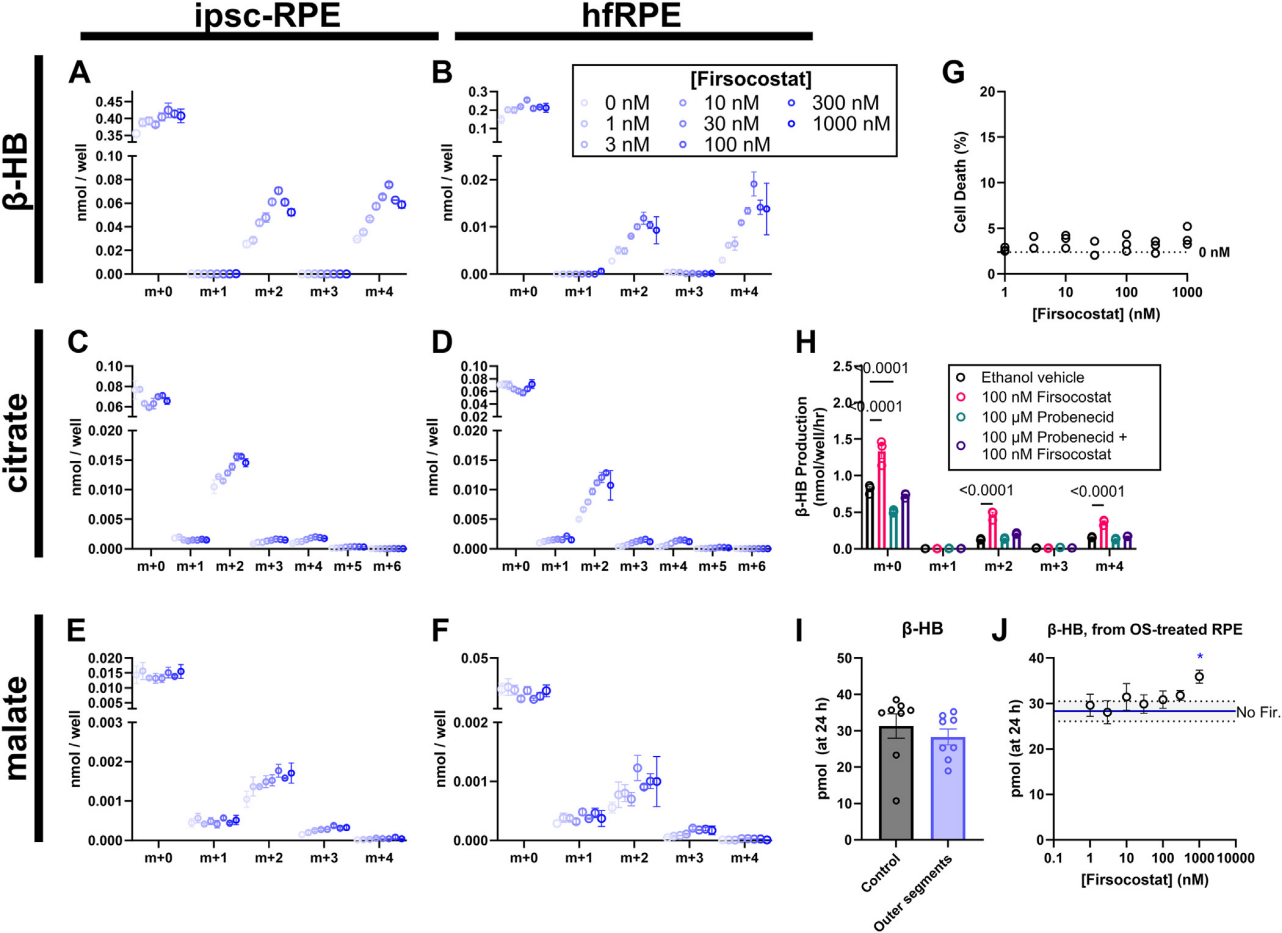
**Firsocostat increases fatty acid oxidation in cultured human RPE cells.** iPSC-RPE cells (*A*, *C*, and *E*) and human fetal RPE cells cultured on a 96-well plate (*B*, *D*, and *F*) were supplied serum-free medium supplemented with 50 μM ^13^C_16_-palmitate and 0, 1, 3, 10, 30, 100, 300, or 1000 nM Firsocostat (n = 3) for 6 h. We collected medium to determine ^13^C labeling on β-HB (*A* and *B*), citrate (*C* and *D*), malate (*E* and *F*). Each isotopolog of a given metabolite is displayed separately. *Darker blue* circles correspond to higher concentrations of Firsocostat. *G*, none of the concentrations of Firsocostat used significantly increased cell death, measured in the same experiment by LDH release into culture medium (n = 3). *H*, probenecid, a nonselective inhibitor of organic anion transport proteins, inhibits the Firsocostat-mediated increase in β-HB labeling by preventing import of the drug over a 6 h period (n = 4). iPSC-RPE cells were cultured on a 96-well plate for 4 weeks and treated with vehicle, outer segments, or outer segments and 0, 1, 3, 10, 30, 100, 300, or 1000 nM Firsocostat (n = 8). *I*, outer segments do not alter β-HB content in culture medium, though (*J*) β-HB levels are increased at the highest tested concentrations of Firsocostat (n = 8). The *red* line and shaded area indicate the mean level of β-HB in medium with outer segments but without Firsocostat, ± the SEM.

RPE-choroid tissue contains RPE cells but also a constellation of other cell types (33). RPE cells are considered a top candidate among the cell types that could produce drusen. In culture, RPE cells can produce drusen-like deposits that may be quantified to assess the level of AMD-like pathology (34, 35, 36). We measured the effects of Firsocostat on β-oxidation in human RPE cultures. We treated both iPSC-RPE cells (Fig. 2, *A*, *C*, and *E*) derived from normal subjects and primary human fetal RPE (hfRPE; Fig. 2, *B*, *D*, and *F*) with 1 to 1000 nM Firsocostat in RPE culture medium containing 50 μM ^13^C_16_-palmitate. Over 6 h, Firsocostat increases the accumulation of ^13^C on β-HB (Fig. 2, *A* and *B*), citrate (Fig. 2, *C* and *D*), and malate (Fig. 2, *E* and *F*) that are released into the culture medium. Concentrations of Firsocostat exceeding 100 nM diminished ^13^C labeling relative to 100 nM Firsocostat. The decline in ^13^C labeling is not caused by toxicity, as a 24-h exposure to Firsocostat does not appreciably increase cell death (Fig. 2*G*). Firsocostat is designed to act upon tissues that express organic anion transport proteins (OATPs) (37). These transporters are strongly expressed on hepatocytes and are inhibited by probenecid (38, 39, 40, 41). 100 μM of probenecid abolishes the Firsocostat-mediated increase in the flux of ^13^C from palmitate to β-HB in iPSC-RPE (Fig. 2*H*), suggesting that RPE cells also express a member of the OATP transporter family, which is used to import the drug.

While oxidation of exogenous ^13^C_16_-palmitate may represent the utilization of circulating lipids by RPE, rod outer segments are another physiological source of retina-derived lipid that is taken up by RPE. We provided RPE cells on a 96-well plate with 0.5 ret/ml of isolated bovine outer segments and 0, 1, 3, 10, 30, 100, 300, or 1000 nM Firsocostat for 24 h, then determined β-HB content in the culture medium. Addition of outer-segments themselves does not clearly impact the accumulation of β-HB (Fig. 2*I*). Adding Firsocostat increases the release of β-HB from RPE cells at most by 27% (with 1 μM Firsocostat) (Fig. 2*J*). Firsocostat can stimulate the oxidation of esterified fatty acids in outer segment lipids and consequent formation of β-HB. However, it is more effective at stimulating the oxidation of BSA-conjugated free fatty acids from culture medium.

### Firsocostat decreases the abundance of lipids in RPE cells

**Figure 3:**
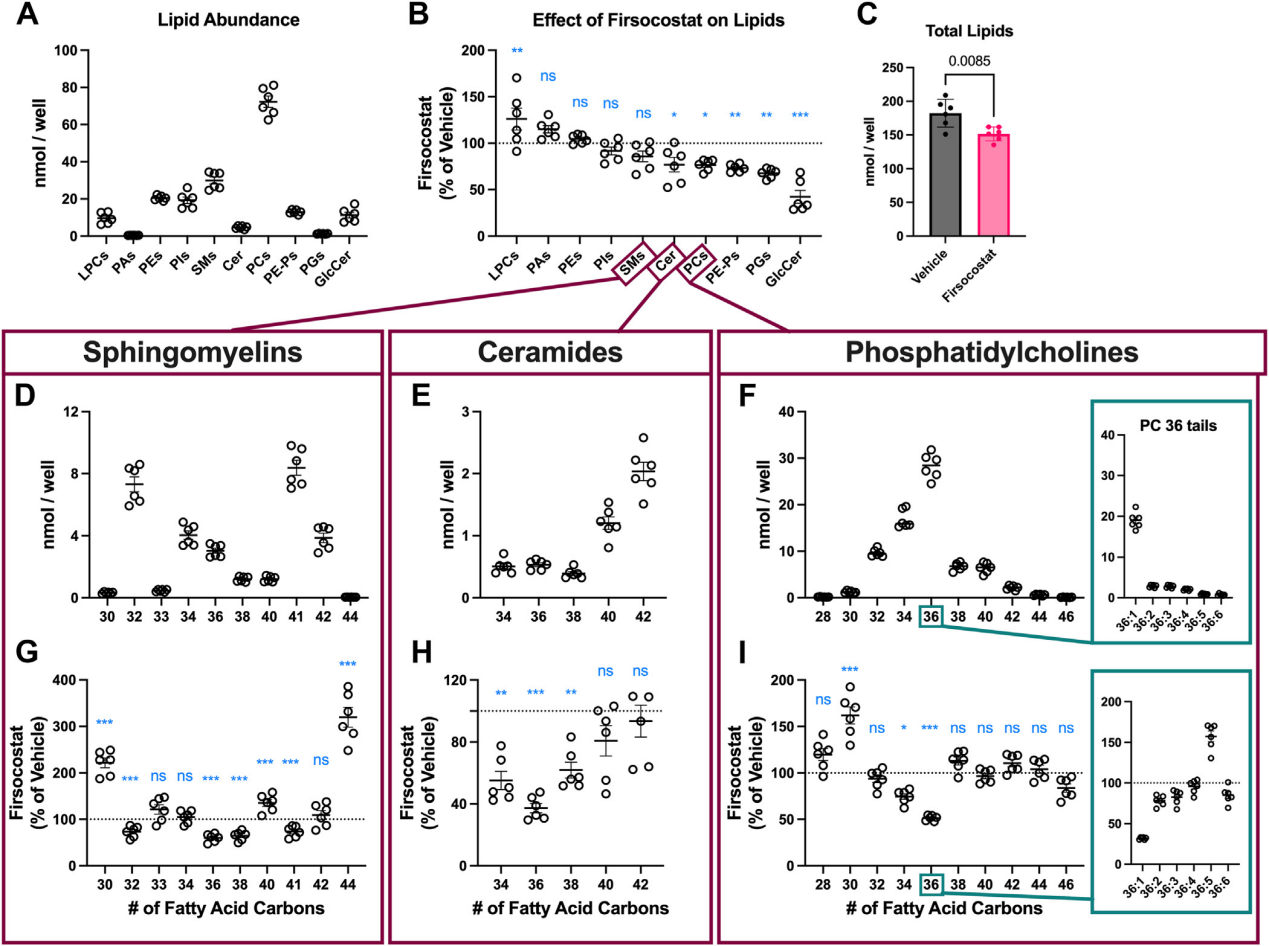
**Firsocostat decreases RPE lipid abundance**. iPSC-RPE cells were cultured for 6 weeks normally, then for an additional 2 weeks in culture medium supplemented with vehicle or 100 nM Firsocostat. Cells were scraped and processed for lipid analysis by HILIC-IM-ToF mass spectrometry to determine the abundance of lysophosphatidylcholines (LPCs), phosphatidic acids (PAs), phosphatidylethanolamines (PEs), phosphatidylinositols (PIs), sphingomyelins (SMs), ceramides (Cer), phosphatidylcholines (PCs), phosphatidylethanolamine plasmalogens (PE-Ps), phosphatidylglycerols (PGs), and glucosylceramides (GluCer). *A*, the abundance of each lipid species in vehicle-treated control samples, (*B*) the change in the abundance of lipid species in RPE cells cultured in Firsocostat, as a percent of vehicle-treated controls. *C*, the total abundance of lipids detected in vehicle and Firsocostat-treated samples. Abundance of (*D*) SMs, (*E*) Cer, and (*F*) PCs, organized by the length of fatty acid tails. The change in the abundance of (*G*) SMs, (*H*) Cer, and (*I*) PCs with 100 nM Firsocostat, quantified as a percentage of abundance in vehicle-treated control RPE cells. Error bars correspond to the mean ± the SEM. Dotted lines in (*B*, and *G–I*) are set at 100% to indicate relative the level of a given lipid in vehicle-treated controls. (n = 6).

Firsocostat increases FAO in mouse RPE-choroid and human RPE cells, but for Firsocostat to affect drusen abundance, it must impact cellular lipid levels. In mouse RPE-choroid, lipid levels are not substantially altered by exposure to Firsocostat for 6 h (Fig. 1, *I* and *J*). A longer-term treatment might allow for changes in fatty acid oxidation to translate to changes in lipid abundance. We cultured human iPSC-RPE from normal donors for 6 weeks, then supplied RPE cells with vehicle or 100 nM Firsocostat for another 2 weeks. We profiled lipids with HILIC-IM-ToF mass spectrometry, using a previously described approach (42, 43). Using known amounts of internal standards for each lipid class, we determined the molar abundance of phosphatidylcholines (PCs), phosphatidylethanolamines (PEs), phosphatidylethanolamine plasmologens (PE-Ps), phosphatidylinositols (PIs), lysophosphatidylcholines (LPCs), phosphatidic acids (PAs), sphingomyelins (SMs), phosphatidylglycerols (PGs), ceramides (Cer), and glucosylceramides (GlcCer). The most abundant lipid class we detected in vehicle-treated RPE were PCs (Fig. 3*A*)

Firsocostat increased the total abundance LPCs and decreased the abundance of Cer, PCs, PE-Ps, PGs, and GlcCer (Fig. 3*B*). Total levels of PAs, PEs, PIs, and SMs were not significantly altered by Firsocostat (Fig. 3*B*), though when the molar amount of all lipids are summed irrespective of class, Firsocostat causes a ∼17% decrease in lipid content (Fig. 3*C*). We hypothesized that even when the total abundance of a lipid class does not change, fatty acids tails will be affected in a chain length-specific manner. We quantified the molar abundance of each chain length by summing individual lipid tails that had different numbers of unsaturated bonds (Fig. 3, *D*–*F*). The most abundant fatty acid tail lengths differed in each class. In SMs, the most abundant tails were 32 and 41 carbons long (Fig. 3*D*). Cer were dominated by longer chain FA tails, with C40 and C42 tails being the most abundant (Fig. 3*E*). On PCs, FA tails between 32 to 40 were most abundant (Fig. 3*F*), particularly C36. Of the fatty acid chain lengths in C36, C36:1 is by far the most abundant (Fig. 3*F*, turquoise box). In each of these lipid classes, Firsocostat decreases in the abundance of FA tails between C32 to 38 (Fig. 3, *G*–*I*). For PCs, this was highly impactful because the most abundant PC fatty acid tail lengths align with the tail lengths that are altered the most by Firsocostat. The majority of this change can be accounted for by a single fatty acid chain, C36:1 (Fig. 3*I*, turquoise box). This result indicates that Firsocostat profoundly affects the lipidome, in part by altering the abundance of PC 36:1. A more detailed depiction of the effect Firsocostat has on PC abundance is available as Fig. S3.

### Firsocostat remodels RPE cell lipid droplet distribution

**Figure 4:**
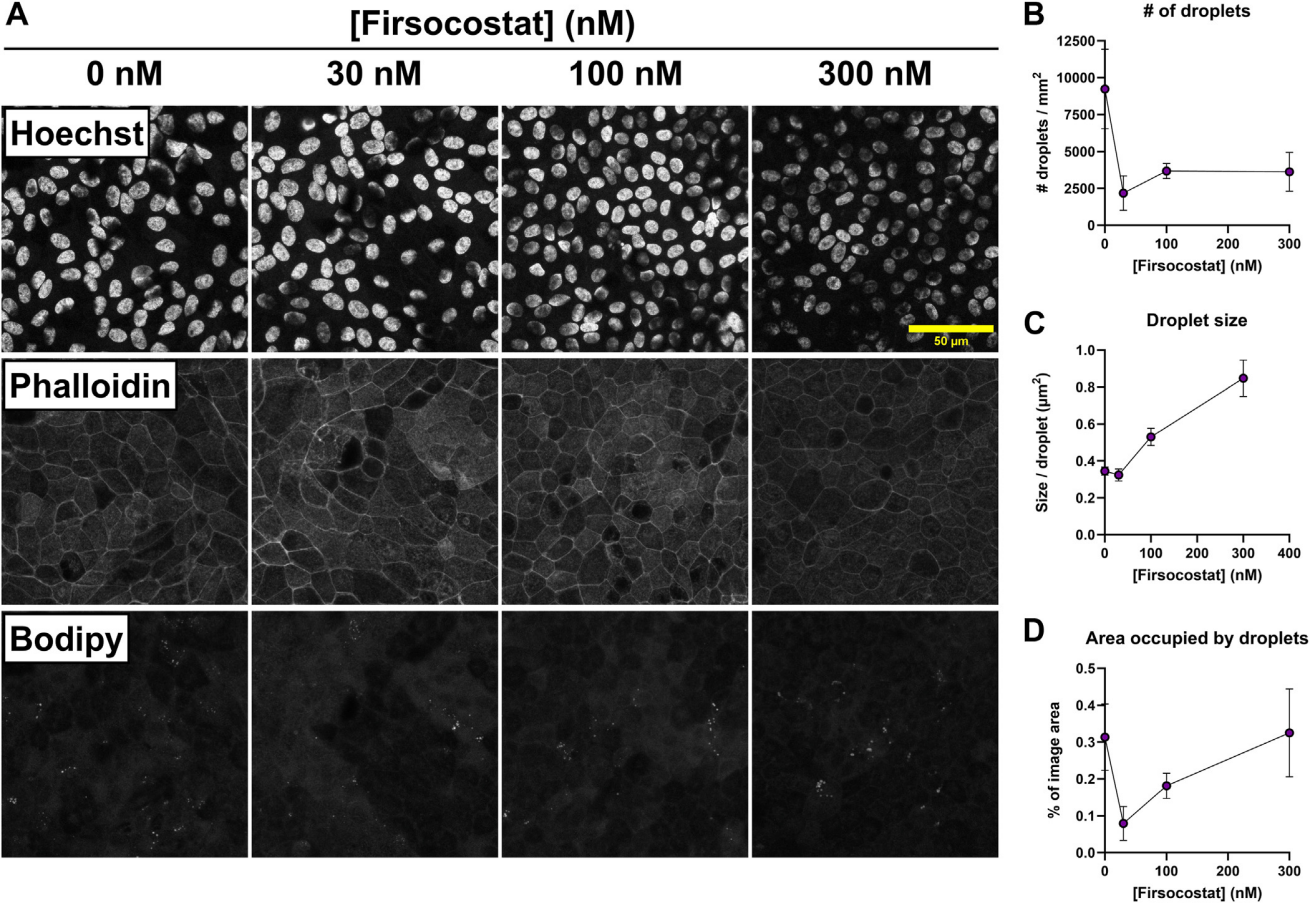
**Firsocostat remodels intracellular lipid droplets.** iPSC-RPE cells were cultured for 6 weeks normally and for an additional 4 weeks supplemented with vehicle, 30 nM, 100 nM, or 300 nM Firsocostat. At 10 weeks, cells were also supplied with 10% human serum apically as a lipid load. Cells were given 10% human serum for 48 h, washed, and fixed in 4% paraformaldehyde. Nuclei, F-actin (cell boundaries), and lipids were labeled using Hoechst-33352, phalloidin-568, and BODIPY-493/503. *A*, shows example single-channel Z-projected images of labeled cells at each concentration of Firsocostat. Z-projected bodipy-493/503 images were quantified in ImageJ. Scale bar (*yellow*) is 50 μm long. The quantification reports (*B*) lipid droplet number per image, (*C*) mean lipid droplet size, and (*D*) the percentage of an image area occupied by lipid droplets (n = 4–5).

**Figure S1:**
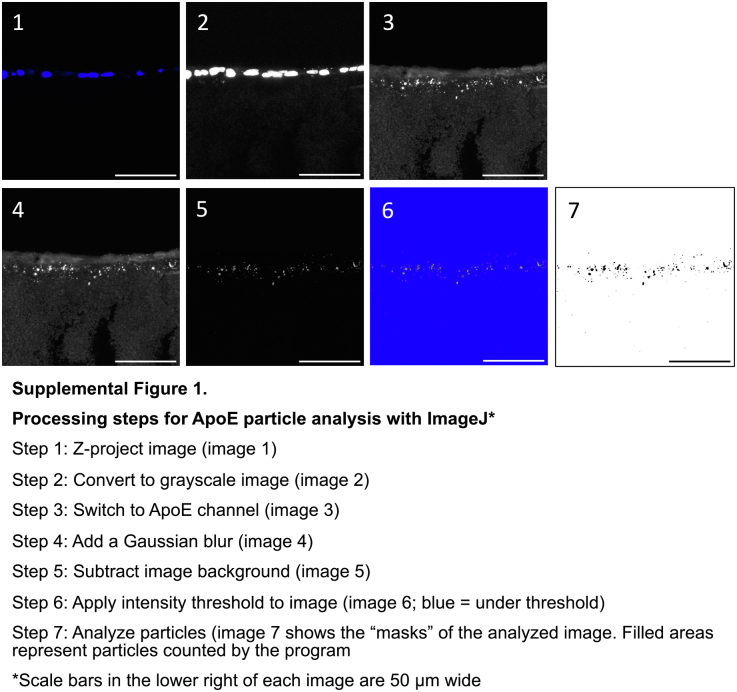


The lipidomics data primarily shed light on the abundance of polar lipids. We tested the hypothesis that Firsocostat could decrease neutral lipid stores in RPE. Neutral lipids can be stored intracellularly in lipid droplets. We cultured control iPSC-RPE cells for 6 weeks under normal conditions, then another 4 weeks either in vehicle alone, 30 nM, 100 nM, or 300 nM Firsocostat. We then increased the intracellular lipid load by adding 10% human serum to their medium, which can increase intracellular lipid content (35, 36). Two days after treatment with serum, the cells were washed and then fixed in 4% paraformaldehyde. Cells on filters were stained *en face* with markers that label nuclei (Hoechst-33258), cell boundaries (F-actin; phalloidin-568), and neutral lipids (bodipy-493/503). Neutral lipids localized to round structures consistent with intracellular lipid droplets (Fig. 4*A*). We quantified the features of these droplets (size, amount, area occupied) using ImageJ (see Fig. S1 for image analysis strategy) and found that Firsocostat consistently lowers the total number of droplets (Fig. 4*B*). Surprisingly, above 30 nM, Firsocostat increased the average lipid droplet size (Fig. 4*C*), so the percentage of each image occupied by lipid droplets is lowered at 30 nM but begins to increase at 100 nM. At 300 nM Firsocostat, the area occupied by lipid droplets is similar to that of vehicle controls, though the lipids occupying the imaging area are in much larger droplets (Fig. 4*D*). These data demonstrate that Firsocostat modifies lipid distribution.

### Firsocostat-dependent effects on glucose metabolism

**Figure 5:**
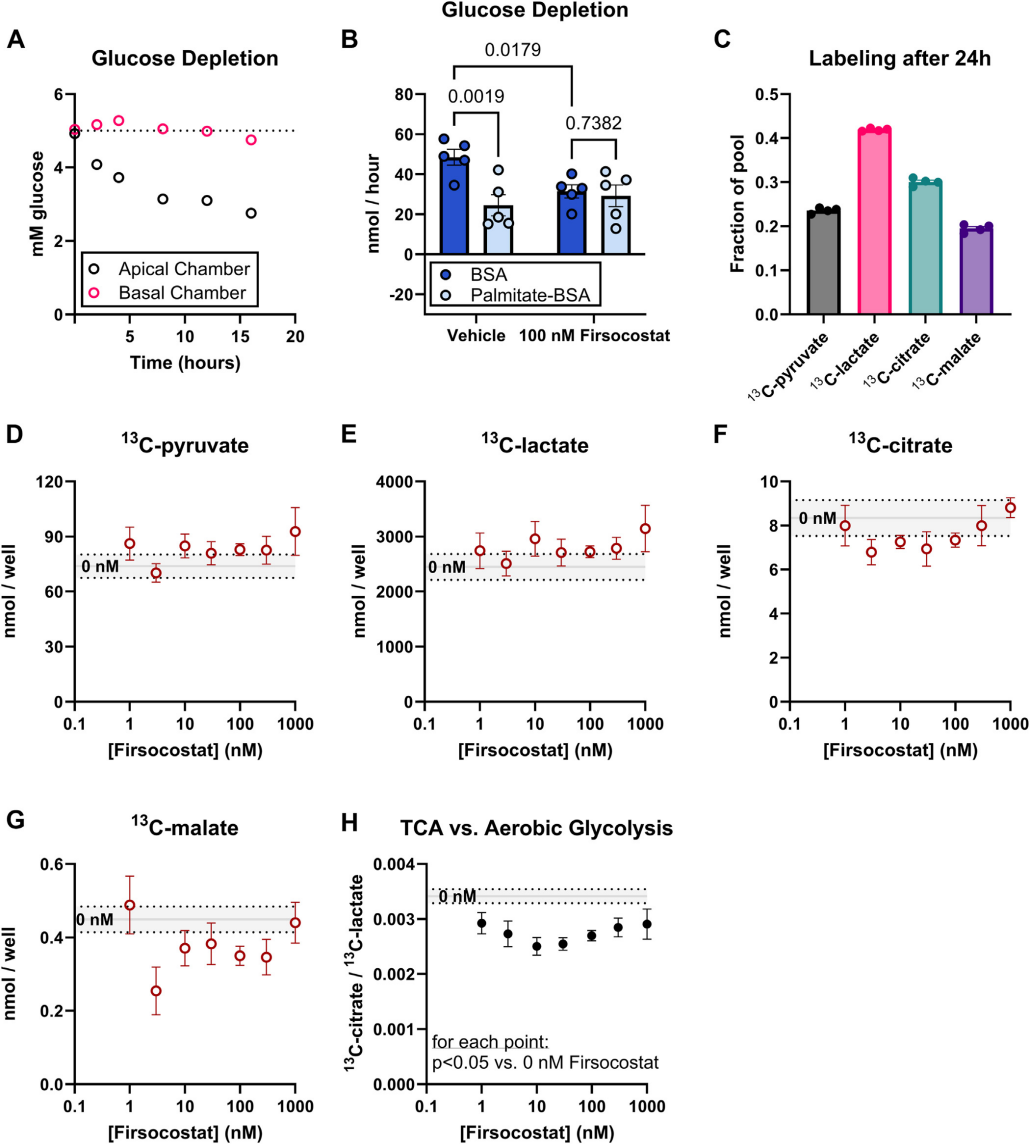
**Firsocostat decreases glucose utilization by the TCA cycle**. iPSC-RPE cells cultured on transwell filters were supplied with fresh serum-free RPE culture medium containing vehicle and no palmitate, vehicle and 300 μM palmitate, 100 nM Firsocostat and no palmitate, or 100 nM Firsocostat and 300 μM palmitate. Medium was sampled from apical and basal chambers at 0, 2, 4, 8, 12, and 16 h, and glucose concentration was assessed enzymatically from samples. *A*, up to 16 h after the medium change, glucose is depleted from the apical chamber but not recognizably changed in the basal chamber. *B*, the rate of glucose consumption by 2 h in the presence or absence of palmitate and Firsocostat is quantified for the apical chamber. To determine how glucose is being used in the presence of Firsocostat, serum-free Miller medium was supplemented with 5 mM ^13^C_6_-glucose and 0, 1, 3, 10, 30, 100, 300, 1000 Firsocostat (n = 4). Medium was collected after 24 h, and we assessed ^13^C labeling of metabolites released by RPE (*C)* in vehicle-only samples, glycolytic and TCA cycle intermediates are well-labeled with ^13^C (maximum possible labeling is 50% because only 50% of glucose is ^13^C_6_). We determined the effect of Firsocostat concentration on all ^13^C-labeled isotopologs of (*D*) pyruvate, (*E*) lactate, (*F*) citrate, (*G*) malate, and (*H*) the ratio of labeled citrate to labeled lactate. For all figures, the *gray* line indicates vehicle-treated controls, and *dotted* lines are the upper and lower bounds of the vehicle mean ± SEM.

Stimulation of FAO by Firsocostat releases more energy from fatty acids. If cellular energy demand is unchanged by Firsocostat, there could be a compensatory decrease in activity of pathways that release metabolic energy from other fuels. In other tissues, FAO can inhibit glucose consumption by a mechanism known as the Randle cycle (44). We determined whether enhancing FAO either by increasing fatty acid levels in culture medium or by treating medium with Firsocostat can alter the rates of glycolysis or glucose-dependent Krebs cycle activity in iPSC-RPE cells. We incubated cells in vehicle or 100 nM Firsocostat, each with either 300 μM palmitate-BSA or equimolar BSA, then determined glucose uptake from the apical or basal chambers. The rate of glucose depletion in the basal chamber is much slower than the rate in the apical chamber (Fig. 5*A*), likely because the filter impedes diffusion of glucose from the basal medium to the basal surface of the RPE cells.

Figure 5*A* shows how glucose is depleted from medium by RPE following a medium change. Fig. S4, *A* and *B* respectively show that the rate of glucose depletion over 8 h, but when we quantified the glucose depletion rate between different time points, we found that glucose depletion from medium was not linear with time (Fig. S4*C*). We therefore show rates of glucose depletion during the first 2 h (Fig. 5*B*). The net amount of glucose consumed during that period is diminished by either Firsocostat or palmitate, but no more so by the combination of both (Fig. 5*B*). Because glucose consumption was not consistently changed during the time that palmitate or Firsocostat were in solution, further studies that focus on this observation will be required to determine if the initial slowing of glucose consumption is related to the Randle cycle.

We also probed medium from RPE cells supplied with 50% labeled ^13^C_6_-glucose for 24 h. ^13^C_6_-glucose strongly labels lactate, pyruvate, citrate, and malate in RPE culture medium (Fig. 5*C*), so we quantified ^13^C labeling of these metabolites to report changes in how glucose is used. On average, cultures treated with Firsocostat made more ^13^C-labeled pyruvate (Fig. 5*D*) and lactate (Fig. 5*E*) while making less citrate (Fig. 5*F*) and malate (Fig. 5*G*). The variation between samples is greater than the effect size, so none of these effects are statistically significant. We normalized levels of ^13^C-labeled citrate to levels of ^13^C-labeled lactate from the same sample, thus quantifying the ratio of carbons from glucose taking the Krebs cycle route versus aerobic lactate production. This normalization reduces sample-to-sample variation and revealed a small, statistically significant influence of Firsocostat on glucose utilization (Fig. 5*H*). Together the data show that Firsocostat does not substantially alter the rate of glucose consumption, but it slightly diminishes glucose input into the Krebs cycle relative to lactate formation. This implies that the Krebs cycle may compensate for increased FAO by slightly diminishing Krebs cycle input from glucose.

### Firsocostat slows the secretion of drusen-promoting lipoproteins by the RPE

**Figure 6:**
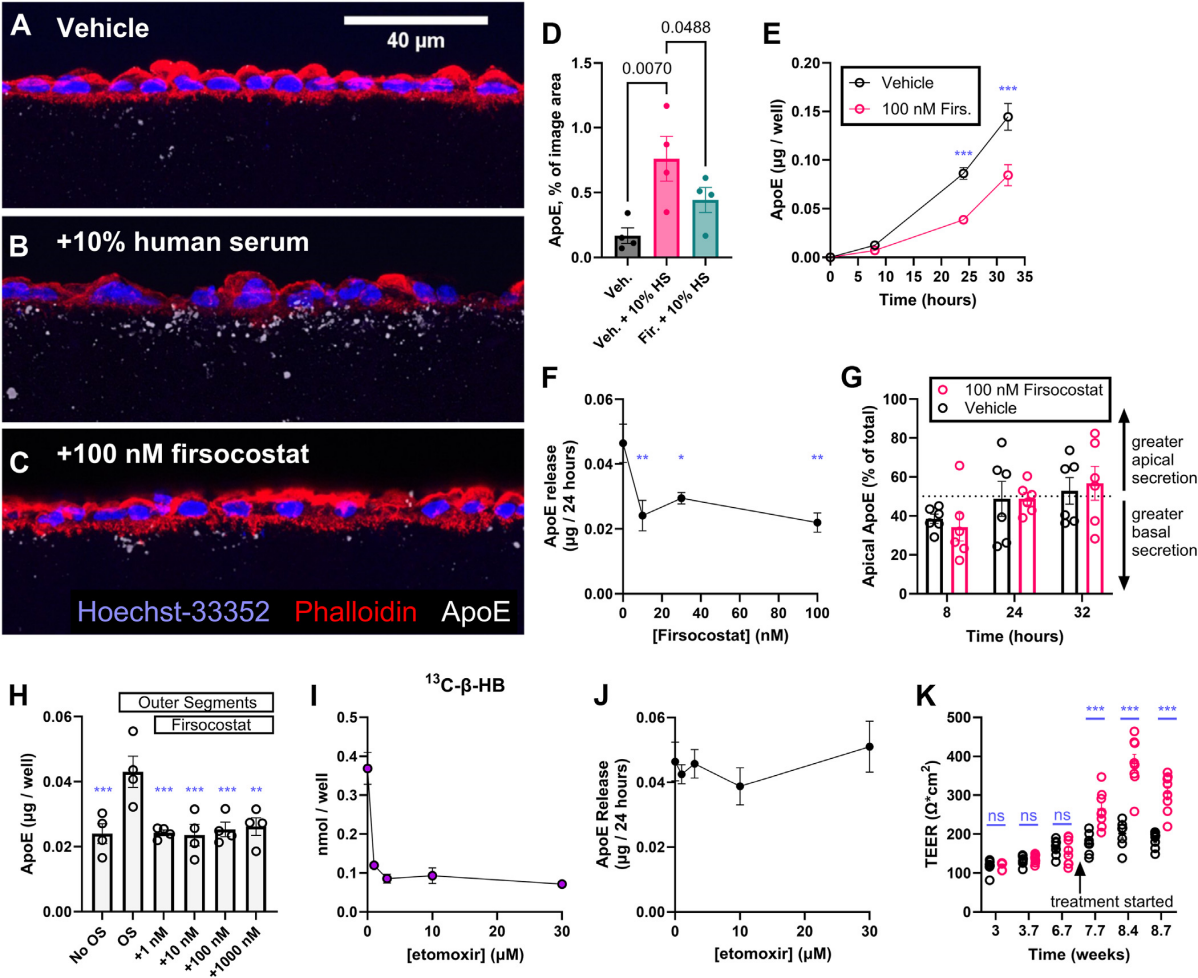
**Firsocostat decreases extracellular accumulation of ApoE.** iPSC-RPE were grown on mixed cellulose ester membranes for 8 weeks, then the apical chamber was supplied either with (*A*) medium containing vehicle, (*B*) vehicle and 10% human serum (v/v), or (*C*) 100 nM Firsocostat and 10% human serum. Firsocostat was provided to both apical and basal chambers, and cells were incubated for 48 h. Cells were fixed in 4% paraformaldehyde and labeled to visualize nuclei (Hoechst-33258, *blue*), F-actin (phalloidin, *red*), and ApoE (*white*). Human serum causes formation of ApoE puncta in the membrane below RPE cells. Scale bar (*white*) is 40 μm long. *D*, Firsocostat decreases the area of the membrane occupied by ApoE puncta (n = 4). *E*, in serum-free miller medium, RPE cells release ApoE over time, and 100 nM Firsocostat decreases the rate of ApoE release (sum of ApoE in apical and basal chambers, n = 6). *F*, over a 24-h period, 10 nM, 30 nM, and 100 nM Firsocostat all decrease ApoE release to a similar extent (n = 4). *G*, the y-axis represents the percentage of total secreted ApoE (basal + apical) that we detected on the apical side. ApoE release is not polarized to the apical or basal side, and Firsocostat does not change the polarity of ApoE release (n = 6). *H*, bovine outer segments (0.5 retinas/ml), supplied for 24 h, significantly increase ApoE release by iPSC-RPE cells, and this release is significantly diminished in the presence of 1, 10, 100, or 1000 nM Firsocostat. *I*, twenty-four hours in etomoxir decreases the ability of ^13^C_16_-palmitate to form ^13^C-βHB, yet (*J*) the etomoxir-mediated decrease in fatty acid oxidation does not alter ApoE release from the same samples(n = 4). *K*, when provided to mature RPE (after 6.7 weeks in culture), Firsocostat increased trans-epithelial electrical resistance (TEER) (n = 8). ∗*p* < 0.05, ∗∗*p* < 0.01, ∗∗∗*p* < 0.001.

Since RPE lipoproteins may contribute to drusen, we tested whether Firsocostat influenced the accumulation of lipoprotein particles in Bruch’s membrane. Lipid and apolipoprotein E (ApoE) form deposits in the filter on which iPSC-RPE cells are grown. These deposits, while formed in culture resemble drusen deposits from AMD patients (34, 35). Human serum stimulates the accumulation of ApoE-containing particles in these filters (34). We supplemented culture medium in the apical chamber with 10% human serum for 48 h with or without Firsocostat, washed the RPE cells, and then fixed them in 4% paraformaldehyde. We immunolabeled cryosections of these filters to detect ApoE within the filter (Fig. 6, *A*–*C*). Ten percent of human serum increases the cross-sectional area of the filter occupied by ApoE deposits (Fig. 6, *A*, *B*, and *D*). 100 nM Firsocostat blunts the effect of 10% human serum so that ApoE deposits occupy a smaller area of the filter (Fig. 6, *B*–*D*). We confirmed this finding with iPSC-RPE cultures treated with or without 100 nM Firsocostat by using an ELISA assay to quantify the release of ApoE into the culture medium. Firsocostat significantly diminishes the release of ApoE (Fig. 6*E*). Our results show that Firsocostat can influence not only FAO but also intracellular lipid handling and lipoprotein release. The effects on lipoprotein release are consistent across a range of Firsocostat concentrations (Fig. 6*F*) and do not affect the polarity of ApoE release to the apical *versus* basal chamber (Fig. 6*G*). Our findings imply that increasing FAO deprives RPE of sufficient lipid to support the assembly and export of drusen-promoting lipoprotein particles. We tested the ability of Firsocostat to limit ApoE export following exposure to another physiological lipid source, rod outer segments. We measured ApoE levels in the same medium samples that we had used to determine whether Firsocostat alters outer segment–associated β-HB release (Fig. 2, *I* and *J*). RPE cells were given vehicle, outer segments, or outer segments and 1, 10, 100, or 1000 nM Firsocostat for 24 h. Outer segments almost double ApoE release, and this effect is blunted at all concentrations of Firsocostat used (Fig. 6*H*).

Since stimulating FAO with Firsocostat suppresses lipoprotein release, we asked whether suppressing FAO stimulates lipoprotein release. We treated RPE cells with 50 μM ^13^C_16_-palmitate-BSA and increasing concentrations (0, 1, 3, 10, 30 μM) of the CPT inhibitor etomoxir. Etomoxir inhibits the oxidation of palmitate by iPSC-RPE (Fig. 6*I*) but does not alter ApoE release (Fig. 6*J*). This suggests that either the rates of FAO alone do not regulate ApoE release or RPE normally releases ApoE near its maximum capacity. Long-term Firsocostat treatment also improves tight junction formation, as RPE cell transepithelial electrical resistance (TEER) increases significantly after treatment with Firsocostat (Fig. 6*K*).

Lipoproteins may be assembled within RPE cells from endogenous sources of fatty acids, cholesterol, and ApoE and then exported. Other types of lipoproteins may pass through the RPE from the circulation on the basolateral side to the retina on the apical side and be remodeled along the way (45, 46). Firsocostat treatment of RPE cells could affect the remodeling of those lipoproteins. ApoA1 associates with high-density lipoproteins (47), while ApoB100 associates with low density lipoproteins (48). We evaluated the effects of Firsocostat on lipoprotein transport across the RPE by providing iPSC-RPE on transwell filters with 10% human serum in the basal chamber to mimic the supply of lipoprotein particles that would come from circulation. If Firsocostat remodels lipoproteins, the rate or extent to which they reach the apical chamber would be altered. We found that Firsocostat diminishes the accumulation of ApoA1 in the apical chamber (Fig. S5*A*), whereas it does not influence ApoB100 transport (Fig. S5*B*). Firsocostat does not alter the overall rate of cholesterol transport (both esterified and unesterified; Fig. S5*C*). These data suggest that Firsocostat may affect the transport of plasma-derived HDL lipoproteins to the retina. An important caveat to this result is that Firsocostat also affects TEER (Fig. 6*K*), so if RPE tight junction function is improved with Firsocostat treatment, it could also limit the transport of lipoprotein particles across the RPE monolayer in an unspecific manner rather than causing a lipoprotein-specific effect.

### Firsocostat improves cellular physiology of iPSC-RPE cells derived from a patient with Sorsby’s fundus dystrophy

Sorsby’s fundus dystrophy (SFD) is a severe inherited retinal disorder with clinical and histological characteristics that overlap with AMD. SFD patients have a thicker Bruch’s membrane, which accumulates large, lipid-rich sub-RPE deposits (15, 49) that bear similarity to drusen in AMD. SFD is caused by mutations in an extracellular matrix metalloprotease inhibitor TIMP3 (50), but the relationship between lipid pathology and the TIMP3 mutations is unclear.

**Figure 7:**
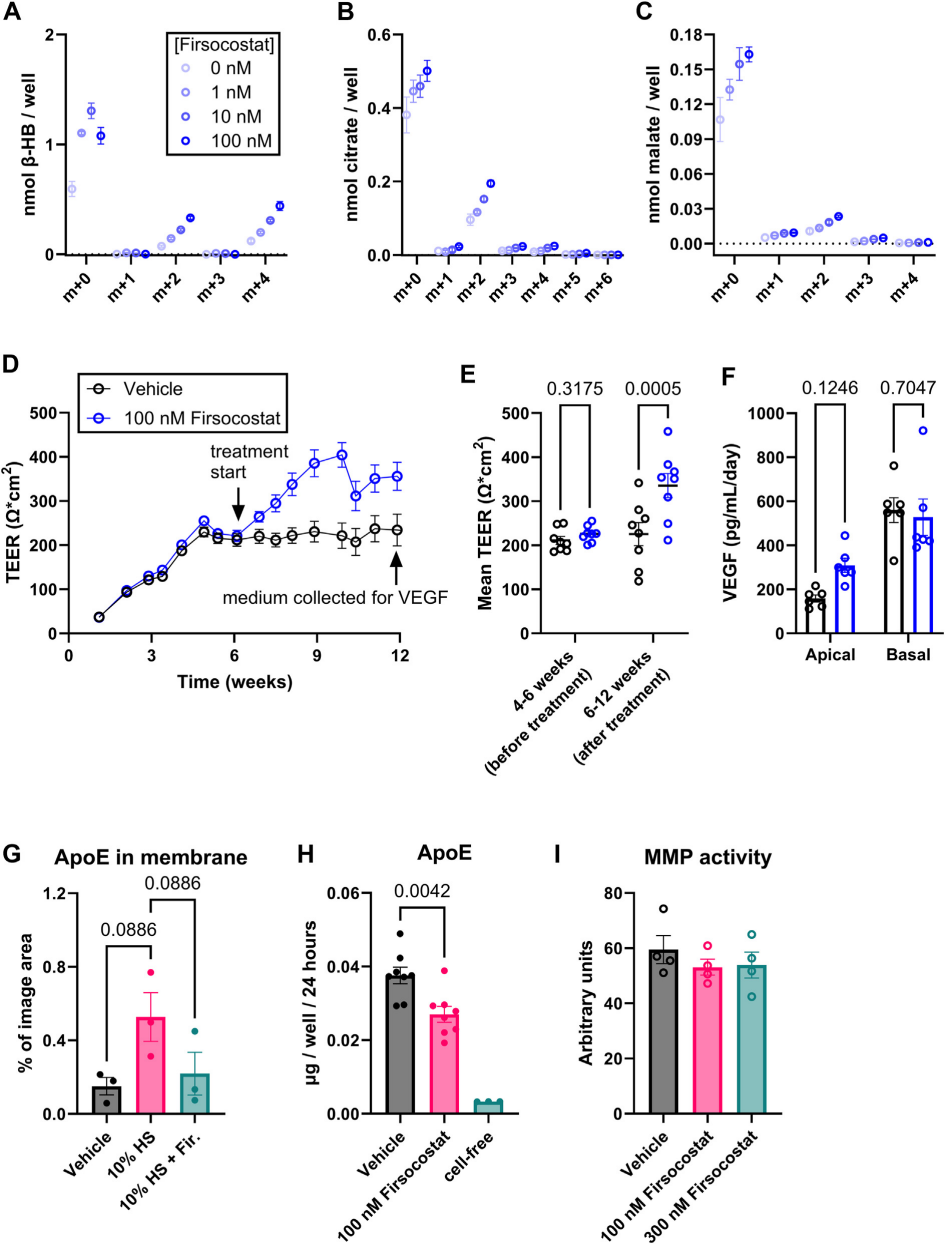
**Firsocostat increases fatty acid oxidation and enhances barrier function in iPSC-RPE from donors with Sorsby’s fundus dystrophy.** iPSC-RPE cells from donors with SFD were cultured on a 96 well plate. At 6 weeks in culture, cells were provided with 50 μM ^13^C_16_-palmitate and 0 nM, 1 nM, 10 nM, or 100 nM for 8 h (n = 4). Following the incubation, we collected culture medium and determined ^13^C labeling on (*A*) β-HB, (*B*) citrate, and (*C*) malate. Each of these intermediates is downstream of fatty acid oxidation. *D*, RPE also was cultured on polyester filters, either with vehicle, 100 nM Firsocostat, or 300 nM Firsocostat. *E*, as with iPSC-RPE from normal donors, 100 nM Firsocostat increases TEER (n = 8). *F*, release of VEGF over a 24-h period by vehicle- and Firsocostat-treated SFD-iPSC-RPE cells is polarized such that more is released to the basal side, but apical VEGF release is selectively increased in cells treated with 100 nM Firsocostat (n = 6). *G*, SFD-RPE were treated for 48 h, either with fresh medium or 10% human serum (apical) and vehicle or 100 nM Firsocostat (both chambers). SFD cells on filters were fixed, sectioned, and immunolabeled for ApoE. The area occupied by ApoE was quantified (n = 3). *H*, ApoE levels were determined in apical medium from SFD RPE treated with vehicle or 100 nM Firsocostat for 24 h (n = 8). *I*, matrix metalloproteinase (MMP) activity in medium was quantified in medium from human RPE cells.

In iPSC-RPE generated from an SFD patient, Firsocostat stimulates the oxidation of 50 μM ^13^C_16_-palmitate-BSA to make ^13^C-β-HB (Fig. 7*A*), ^13^C-citrate (Fig. 7*B*), and ^13^C-malate (Fig. 7*C*), which accumulate in culture medium after 8 h. As it does with iPSC RPE from normal donors, addition of Firsocostat to SFD-iPSC-RPE cultures increases TEER for at least 6 weeks (Fig. 7*D*). We quantified the steady-state TEER for each well before and after application of 100 nM Firsocostat. Figure 7*E* shows that 100 nM Firsocostat increases TEER. After 12 weeks, we checked cell polarity by quantifying VEGF released from RPE to the apical and basal chambers. SFD RPE, like normal RPE, secretes more VEGF into the basal chamber (15) (Fig. 7*F*). 100 nM Firsocostat increases apical VEGF release while still maintaining the overall basal polarity of VEGF secretion. High levels of VEGF could stimulate pathologic neovascularization in SFD but VEGF is also a trophic factor. It can promote Müller glia, photoreceptor, and inner retinal neuron survival without impacting blood vessel integrity (51, 52). The stimulation by Firsocostat of VEGF release from RPE cells is small and not statistically significant (Fig. 7*F*).

We measured the deposition of ApoE in the filter after providing SFD cells with 10% human serum. ApoE deposition increases on average with serum and decreases on average with 100 nM Firsocostat, but the change in ApoE accumulation is not statistically significant (*p* = 0.09; Fig. 7*G*). As with iPSC-RPE from normal patients, 100 nM Firsocostat significantly inhibits the release of ApoE from SFD patient-derived RPE (Fig. 7*H*). The impact of Firsocostat on SFD pathology may be restricted to its influence on the accumulation of fatty acids and lipoproteins. Firsocostat does not alter the activity of matrix metalloproteinases secreted by SFD RPE (Fig. 7*I*). Together our data shows that Firsocostat can stimulate FAO, decrease lipoprotein release, and enhance tight junction integrity in SFD RPE cells.

## Discussion

There are no FDA-approved treatments for AMD or SFD that directly affect lipid metabolism, yet the need for drugs that target lipid and lipoprotein accumulation has been acknowledged by leaders in the field (16, 17). Our investigation of ACC inhibition begins to address that need. Our studies show that Firsocostat accelerates fatty acid oxidation in mouse RPE-choroid tissue and in cultured human RPE cells.

The Firsocostat-dependent increase in fatty acid oxidation from palmitate-BSA appears to be stronger than the increase in fatty acid oxidation from outer segments. The outer segments were recognized by RPE cells and stimulate Apolipoprotein release from RPE. Yet it is unclear whether outer segment fatty acids were oxidized within RPE. Unlike in prior reports, we did not see an increase in β-HB release when we supplied outer segments to RPE cells (53). Our results could differ from previous findings for one of several reasons, including differences in RPE cell behavior (iPSC-RPE *versus* fetal RPE or ARPE-19 cells), RPE environment (medium composition and volume), or in the type of biochemical assay used to assess β-HB levels (54).

In addition to affecting fatty acid metabolism, stimulating FAO in RPE cells slows glucose consumption, and the carbons from glucose are partially diverted to make lactate. Unlabeled acetyl-CoA derived from FAO may compete with ^13^C-labeled acetyl-CoA from glycolysis to lower the ratio of ^13^C-citrate to ^13^C-lactate (Fig. 5*I*). If this is true, increasing energy supplied from one metabolic process could decrease energy supplied from the other.

Firsocostat's effect on fatty acid oxidation influences lipid and lipoprotein metabolism in RPE cells. Most lipid classes are unchanged or significantly decreased when RPE cells are cultured in Firsocostat. The exception is lysophosphatidylcholines, a precursor or product of phosphatidylcholines with one less fatty acid tail. An increase in the abundance of LPCs may reflect a decrease in synthesis or an increase in the degradation of phosphatidylcholines, which themselves are decreased with Firsocostat. Cer, PE-Ps, PGs, and GlcCer are also decreased with Firsocostat.

PCs are highly abundant in membranes, present in lipoproteins, and accumulate in drusen (4). A decrease in PC content could decrease the drive for RPE to generate and release lipoprotein-related lipids. In contrast, Cers are not abundant in lipoproteins. They are critical signaling lipids. Their accumulation appears to be negatively correlated with RPE health in several studies. C2 ceramides are toxic to RPE cells (55), particularly when they are poorly polarized (56). Intracellular ceramides accumulate following iron-mediated cell damage and decreases RPE autophagic flux (57, 58). Inhibiting ceramide production may correct abnormal accumulation of endosomes by unhealthy RPE cells (59), suggesting that decreases in ceramide abundance could maintain RPE health. The lipid classes most strongly impacted by Firsocostat are PE-Ps, phosphatidylglycerols, and glucosylceramides. The physiology of these lipids in RPE is poorly defined, and a ripe area for speculation and future research.

Most of the lipid types we quantified possess two fatty acyl tails. The tails that decrease with Firsocostat treatment tend to be <C40, that is, the sum of two tails where at least one fatty acyl tail is <C20. ACC inhibition dis-inhibits CPT and thus should enhance the oxidation of long chain fatty acids (C14-C18). The stronger effect of Firsocostat on fatty acid tails <C40 may suggest that its effects on RPE lipid composition result from its acceleration of fatty acid oxidation rather than a Firsocostat-mediated inhibition of RPE lipid synthesis.

The size and number of lipid droplets is also altered with increasing Firsocostat concentrations. What regulates droplet size and number? Lipid droplets are degraded either gradually with lipases (lipolysis) or en mass in the lysosome (lipophagy) (60, 61). Either process can increase free fatty acid abundance. The process of lipolysis and lipophagy may yield very distinct lipid droplet signatures. Hepatocytes treated with chloroquine are forced to use lipolysis, which results in many small lipid droplets (62). When hepatocytes are given atglistatin to inhibit adipose triglyceride lipase, the major cytosolic lipase responsible for lipid release from lipid droplets, they must use lipophagy to digest droplets. In atglistatin-treated cells, lipid droplets are fewer and larger than controls (62). If ‘preferential’ utilization of lipophagy also increases lipid droplet size in RPE cells, Firsocostat could be increasing the RPE cell’s reliance on lipophagy to digest lipid droplets, though due to the many regulators of lipid droplet size, this is only one possibility of several (63).

In addition to altering lipids levels and distribution, Firsocostat slows the release of ApoE from RPE cells both in the presence or absence of external lipids from photoreceptor outer segments or from human serum. As a consequence of the decrease in ApoE release, fewer ApoE-containing particles are found in the filter substrate on which the RPE cells grow. This is perhaps the most central result in this study, because lipoproteins are released to the extracellular environment, where drusen form. Firsocostat-induced changes to Apolipoprotein release thus reflect the potential of this drug to decrease levels of drusen.

The RPE is an epithelial barrier that prevents unregulated diffusion of circulating metabolites to the retina. Measurements of TEER report this barrier function. In addition to directly affecting fatty acid, lipid, and lipoprotein metabolism, Firsocostat increases TEER. This result, while initially puzzling, correlates well with how barrier function of intestinal epithelial cells is affected by fatty acids. Palmitic acid impairs tight junction integrity and TEER in both cultured enterocytes (64) and intestinal organoids (65). Long chain saturated fatty acids impair energy generation by intestinal epithelial cells and impair TEER by decreasing the expression of transcripts for tight-junction proteins. Similarly, high fat diet increases permeability of the intestine by altering the composition of tight junctions (66). While the mechanism is not yet clear, the impact of fatty acid and lipid composition on plasma membrane biomechanics and on energy production could each potentially play a role in altering retinal pigment epithelial permeability, as in the intestine. Firsocostat may therefore work in to oppose increases in RPE permeability, by decreasing fatty acid synthesis and increasing fatty acid oxidation.

**Figure S2:**
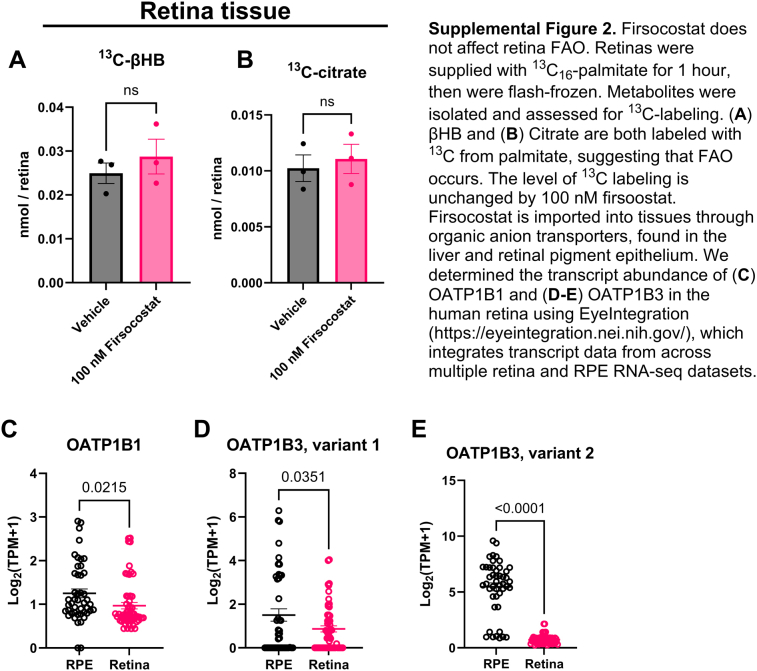


**Figure S3:**
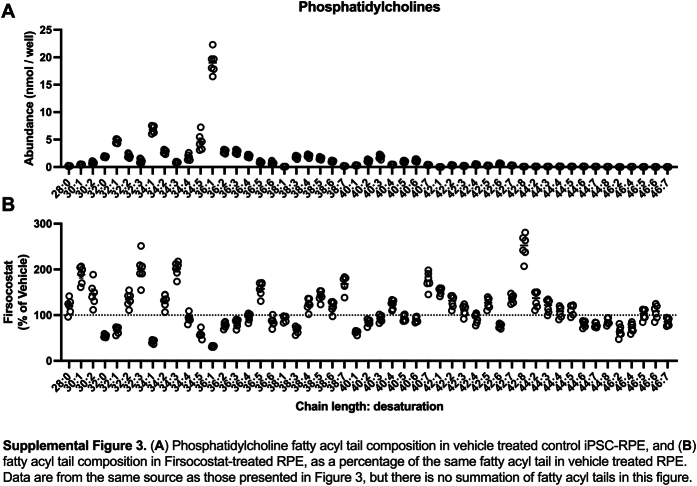


**Figure S4:**
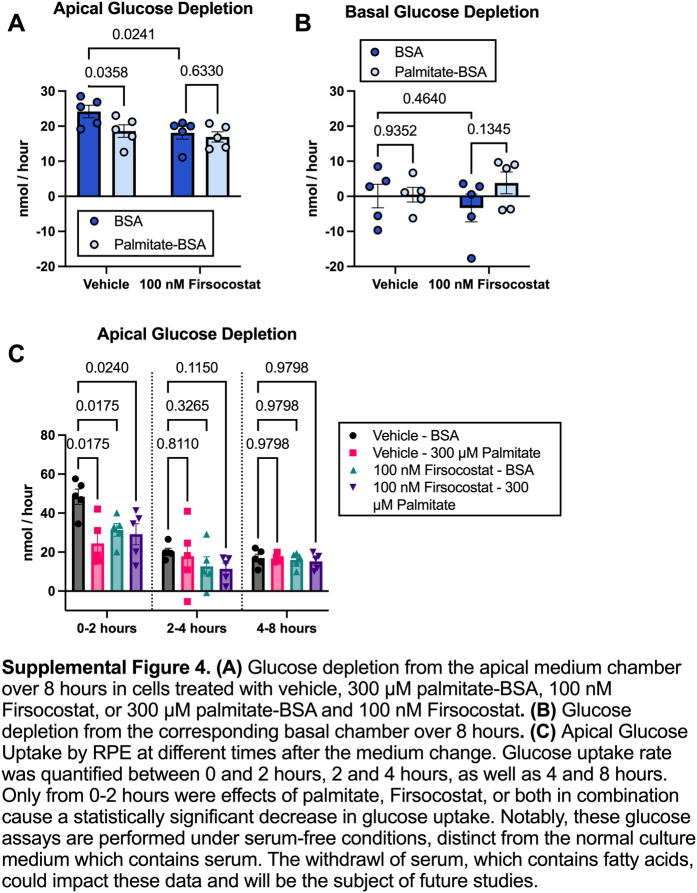


Firsocostat is not necessarily beneficial for the eye. A lower abundance of fatty acids or lipids in photoreceptors could negatively affect outer segment biogenesis and thus retina function. We find that Firsocostat does not affect fatty acid oxidation in retinas. The distribution of OATPs suggests that retinas may not be able to import the drug (Fig. S2). Together these results imply that Firsocostat will not deprive photoreceptors of lipids, though it will be crucial for us to test the impact of Firsocostat in animals directly, to ensure that treatment modalities aimed at increasing fatty acid oxidation do not impair visual function.

**Figure 8:**
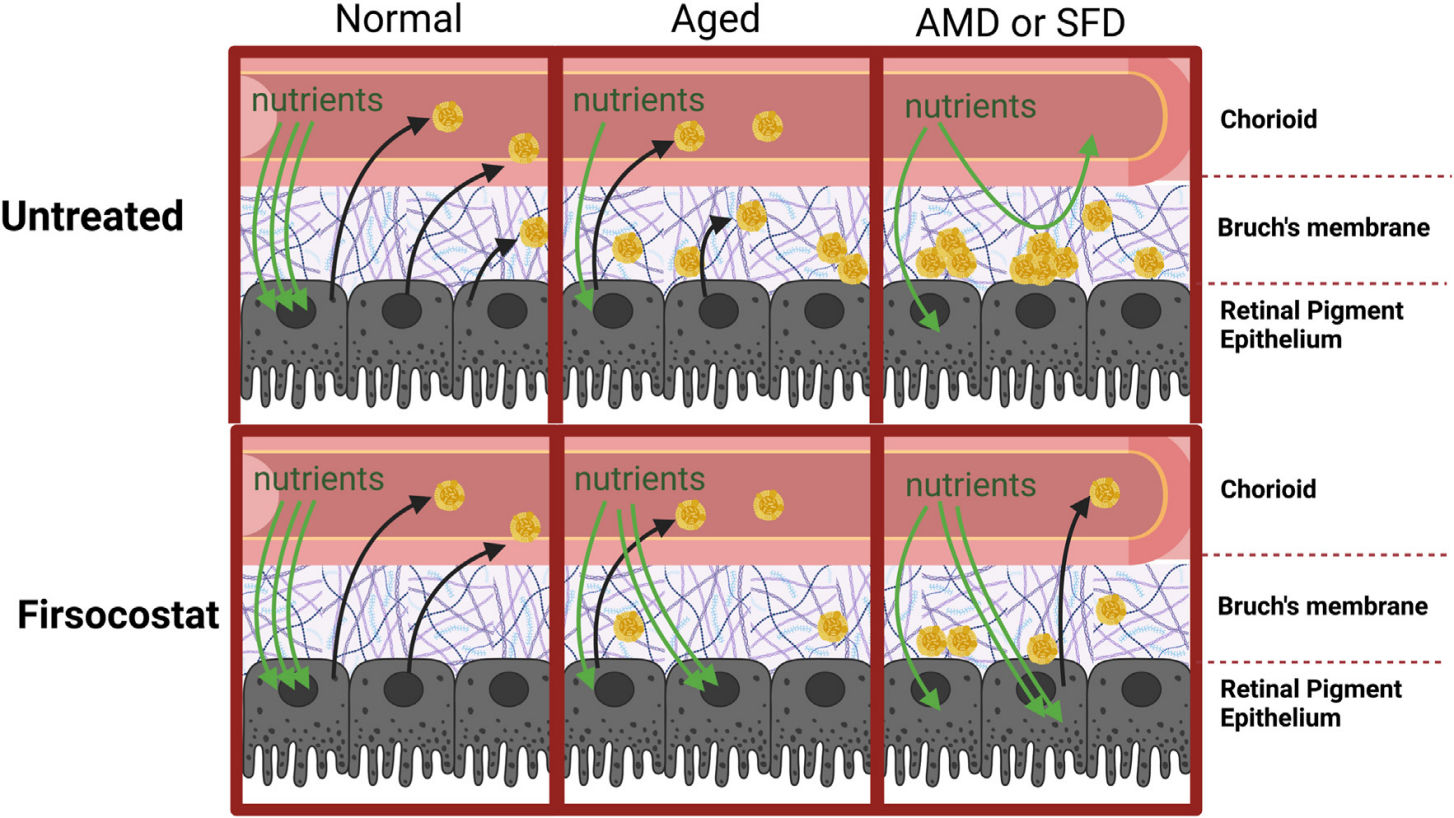
**Diagram illustrating the hypothetical role of Firsocostat may play in lessening AMD or SFD pathology**.

In summary, we have found evidence that Firsocostat limits lipoprotein particles release, likely because it limits the accumulation of intracellular lipids. Our hypothesis for how Firsocostat could treat AMD is depicted in Figure 8. If Firsocostat or other small molecule inhibitors of acetyl-CoA carboxylases can limit the production and release of lipoproteins and slow accumulation of drusen in patients, they could become effective therapeutic agents for AMD.

## Experimental procedures

### Mice

All experiments adhered to our approved IACUC protocol and the ARVO statement for the use of animals in ophthalmology research. For animal experiments, we used 2 to 6 month-old C57BL/6J mice (JAX, stock# 000664) fed ad libitum on a 12-12 light/dark cycle. Mice were euthanized by awake cervical dislocation. The time of day for euthanasia was later than 11 PM, to avoid variability due to peak outer segment phagocytosis. To obtain RPE-choroid tissue for experiments, eyes were first dissected from the head into room temperature Hank’s buffered salt solution (Gibco, 14170120). Eyes were cleared of extraocular muscle and collagenous tissue, then cut along the ora serrata. Cornea, iris, and lens tissue were removed, then the remaining retina and RPE-choroid were teased apart for downstream experiments. This process typically takes ∼3 to 4 min for both eyes from one mouse.

### Measurement of fatty acid oxidation: Mouse RPE-choroid

Freshly dissected retina and RPE-choroid were incubated in KRB buffer containing 5 mM D-glucose, 50 μM [U-^13^C_16_]-palmitic acid (Cambridge isotope labs, CLM-409) complexed to bovine serum albumin at a 6:1 ratio of fatty acid to albumin (67), and vehicle (ethanol) or Firsocostat (Caymen Chemical, 23961). Incubation medium was equilibrated >30 min at 37 °C and 5% CO_2_ prior to the tissue dissection. Tissues were incubated for 1 h at 37 °C and 5% CO_2_, then flash-frozen in liquid N_2_, and stored at −80 °C prior to downstream processing.

### Measurement of fatty acid oxidation: RPE cells

RPE cells were incubated in pH 7.4 Miller medium (68) and 50 μM [U-^13^C_16_]-palmitic acid (Cambridge isotope labs, CLM-409) complexed to bovine serum albumin at a 6:1 ratio of fatty acid to albumin. This buffer was pre-equilibrated at 37 °C, 21% O_2_, and 5% CO_2_ prior to incubations. We changed culture medium on cells, washed them in sterile 1× PBS, and incubated them in medium containing the ^13^C label. To determine metabolite uptake or export rate, we sampled incubation medium at times indicated in the text and figures.

### Metabolite extraction

Metabolites were extracted from tissue or media samples using 80% MeOH, 20% H_2_O, with 10 μM methylsuccinate (Sigma, M81209). The extraction buffer was equilibrated on dry ice, then 100 to 150 μl was added to each sample. Tissues were disrupted by sonication, then incubated on dry ice for 45 min to precipitate protein. Proteins were pelleted at 17,000*g* for 30 min at 4 °C. The supernatant containing metabolites was dried under vacuum at room temperature until dry and stored at −80 °C until derivatization.

### Metabolite derivatization

Dried samples were derivatized by adding 10 μl of 20 mg/ml methoxyamine HCl (Sigma, 226904) dissolved in pyridine (Sigma, 270970) and incubating at 37 °C for 90 min. Samples were further derivatized by adding 10 μl tert-butyldimethylsilyl-N-methyltrifluoroacetamide (Sigma, 394882) and incubating at 70 °C for 60 min.

### Gas chromatography-mass spectrometry

Derivatized samples were injected into an Agilent 7890/5975C GC-MS system. Selected-ion monitoring was used to determine metabolite abundance, as previously described (69). Peaks were integrated using MSD ChemStation E.02.01.1177 (Agilent Technologies), and correction for natural isotope abundance was performed with IsoCor, v1.0 (70). Corrected metabolite signals were converted to molar amounts by comparing metabolite peak abundances in samples with those in a ‘standard mix’ containing known quantities of metabolites we routinely measure. Multiple concentrations of this mix were extracted, derivatized, and run alongside samples in each experiment. These known metabolite concentrations were used to generate a standard curve that allowed for metabolite quantification. Metabolite abundance was normalized to tissue protein concentration, and following this, paired tissues such as retina or RPE-choroid samples from the same mouse were treated as technical replicates and averaged.

### Lipidomics of mouse RPE-choroid

Mouse RPE-choroid was dissected then incubated in KRB supplemented with 50 μM unlabeled palmitate-BSA and 5 mM glucose, at 37 °C and 5% CO_2_ for 6 h. Tissue was flash-frozen in liquid nitrogen. To extract tissue lipids, samples in purified LC/MS-grade water were homogenized in a Bullet Blender with zirconia beads for 15 min and then transferred to glass culture tubes. 25 μl of an isotope labeled internal standard mixture, 575 μl of methyl-*tert*-butyl ether (MTBE), and 160 μl of methanol (MeOH) were added to each sample. Samples were shaken for 30 min at room temperature. 200 μl of H_2_O was added, then samples were centrifuged at 2500*g* for 3 min at room temperature, resulting in the formation of two liquid layers. The upper layer was transferred to a new glass vial using a glass Pasteur pipette. Lipids were extracted again from the lower layer by adding 300 μl MTBE, 100 μl MeOH, and 100 μl of H_2_O, followed by shaking and centrifugation as in the first extraction. The upper layer from the second extraction was pooled with the upper layer from the first extraction and dried under nitrogen. Dried samples were reconstituted in 250 μl of 10 mM ammonium acetate in 50:50 MeOH:dichloromethane. A BCA assay was used to determine protein concentration in sample precipitate. Lipid levels were then normalized to protein content. Lipid and fatty acid species in the extracted tissue samples were measured on a Sciex Lipidyzer mass spectrometry platform as previously described (71). The system consists of Shimadzu Nexera X2 LC-30AD pumps, a Shimadzu Nexera X2 SIL-30AC autosampler, and a Sciex QTRAP 5500 mass spectrometer equipped with SelexION for differential mobility spectrometry (DMS). 1-propanol was used as the chemical modifier for the DMS. Samples were introduced to the mass spectrometer by flow injection analysis at 8 μl/min. Each sample was injected twice, once with the DMS on (PC, PE, LPC, LPE, and SM) and once with the DMS off (CE, CER, DAG, DCER, FFA, HCER, LCER, and TAG). Lipid molecular species were measured using multiple reaction monitoring and positive/negative polarity switching. SM, DAG, CE, CER, DCER, HCER, DCER, and TAG were ionized in positive mode and LPE, LPC, PC, PE, and FFA in negative ionization mode, respectively. Data were acquired and processed using Analyst 1.6.3 and Lipidomics Workflow Manager 1.0.5.0. 440 to 665 lipids were measured across the study set of six samples. All the samples were prepared and analyzed in a single batch. For quality control, a pooled sample was run at the beginning and at the end of the batch, respectively. The median CV was typically ∼5.0%.

### iPSC-RPE and hfRPE culture

The generation and differentiation of normal and SFD iPSC-RPE lines in this manuscript have been described previously (15, 72). Informed consent was obtained from all subjects (University of Washington IRB-approved STUDY00010851). Fetal RPE were obtained from the Birth Defects Research Laboratory at the University of Washington (UW) under an approved protocol (UW5R24HD000836). All experiments were conducted according to the principles expressed in the Declaration of Helsinki.

Below we summarize how differentiated cells were cultured in this study. For the first week in culture, differentiated RPE cells were cultured in MEM-α medium (Invitrogen, 12561-072) supplemented with 5% (v/v) fetal bovine serum (FBS) (Atlanta Biologicals, S11550), 1× non-essential amino acid solution (Invitrogen, 11140-050), 1% (v/v) N1 supplement (Sigma-Aldrich, N6530), 50 U/ml penicillin/streptomycin (Invitrogen, 15070-063), 0.5 mg/L taurine (Sigma-Aldrich, T0625), 40 ng/L hydrocortisone (Sigma-Aldrich, H0396), 1.3 ng/L triiodo-thyronine at (Sigma-Aldrich, T5516), and 10 μM Y-27632 (Selleck chem, S1049). After the first week, FBS concentration was reduced to 1% FBS and Y-27632 was omitted for the rest of the culture period, and cells were cultured in standard miller medium (68). Medium was changed every 2 to 3 days.

A subset of iPSC-RPE and hfRPE cells were plated in sterile tissue culture-treated 96-well plates (Corning, 3603) at 50,000 cells/well (corresponding to data in Figs. 2, 5, *C*–*H*, 6, *F*, *H*–*J*, and 7, *A*–*C*). Cells were cultured for 4 to 6 weeks prior to experiments. Cells were supplied with 200 μl of medium per well. For experiments where cells were incubated in a ^13^C-labeled substrate, culture medium was serum-free but otherwise identical to conventional culture medium. For 96-well plates, medium volume was 200 μl/well, leading to an estimated 0.63 cm apical medium height. When cells were supplied with outer segments, they were bovine outer segments (InVision Bioresources, 98740) provided at a concentration of 0.5 retina/ml. When Firsocostat (Caymen Chemical, 23961) was provided, the same concentration of vehicle (100% ethanol) was provided to each sample. The final concentration of ethanol was 0.1%.

RPE cells in other experiments (Figs. 4, *A*–*D*, 5, *A* and *B*, 6, *A*–*E*, *G*, and *K*, 7, *D*–*I*) were cultured in 24-well plates (Falcon, 353047) on mixed cellulose ester Millicell inserts (Millipore, PIHA01250). Cells were plated at 100,000 cells/insert and cultured 6 to 8 weeks before experimental manipulations. We supplied cells with 400 μl of culture medium to the apical chamber and 600 μl to the basal chamber of each well. Medium height is estimated to be 0.67 cm.

For immunostaining experiments, cells were treated with vehicle or 10% (v/v) normal human serum (Millipore, S1-100 Ml, lot# 2330486) in serum-free media, supplied to the apical chamber at 8 weeks for 48 h. When Firsocostat (Caymen, 23961) was supplied to cells, it was made as a 100× solution in ethanol and diluted in medium. When probenecid (Caymen, 14981) was supplied to cells, it was diluted from a 100× stock in DMSO, and an equal amount of DMSO was also used in the control group.

### Lipidomics of human iPSC-RPE cells

We seeded normal human RPE cells at 600,000 cells/well of a 12-well plate (Greiner, 665165), and cultured them for 6 weeks. We then treated cells with either 0.1% ethanol vehicle or 100 nM ND630 in 1 ml of medium/well for an additional 2 weeks. For lipid extraction, cells were scraped in PBS (300 μl/well, twice) over ice, and PBS was pipetted into a glass vial. Cells were sonicated in ice water for 30 min to ensure disruption of the plasma membrane. 10 μl per sample of a 240 μM internal standard solution was added to each sample. Individual internal standards in the mix are listed in Table 1.

**Table 1:** Internal standards used to quantify lipid abundance

Name	Vendor	Catalog	MW (g/mol)
Phosphatidylethanolamine (15:0/15:0)	Avanti	850704X	663.906
Ceramide (d18:1/17:0)	Avanti	860517P	551.927
Phosphatidylglycerol (15:0/15:0)	Avanti	840446X	716.899
Phosphatidylcholine (15:0/15:0)	Avanti	850350C	705.986
Phosphatidic acid (12:0/12:0)	Avanti	840635X	558.661
Phosphatidylserine (12:0/12:0)	Avanti	840038X	645.738
Sphingomyelin (d18:1/17:0)	Avanti	860585P	717.055
Lysophosphatidylcholine (15:0/0:0)	Avanti	855576C	481.603
Lysophosphatidylethanolamine (13:0/0:0)	Avanti	110696	411.471
Diglyceride (13:0/13:0)	Nu-Chek	D-136	484.7519
Triglyceride (15:0/15:0)	Nu-Chek	T-145	765.2405
d7-Phosphatidylinositol (15:0–18:1)	Avanti	791641C-1 mg	847.116

Samples were extracted with 4 ml of Folch solution (2:1 Chloroform:Methanol) and 1 ml of 0.9% (w/v) NaCl in H_2_O. Lipid extracts were dried, reconstituted in dichloromethane, and stored at −80 °C. On the day of LC-MS analysis, lipids were resuspended in 2:1 acetonitrile:methanol. Lipids were separated by liquid chromatography on a Waters Acuity UPLC system equipped with a HILIC column (2.1 × 100 mm, particle size: 1.7 μm). Traveling wave ion mobility was used to separate lipids by drift time (corresponds to collisional cross section). Time-of-flight mass spectrometry was used to separate lipids by *m/z*. LC solvents, method, and instrument calibration steps are identical to those used in past publications (43). Samples were scanned in both positive and negative modes, and data was processed using Progenesis QI (Nonlinear Dynamics). Lipid identification was performed with a custom python script called LiPydomics (73) and HMDB database searching, to match lipid species by retention time, *m/z*, and collisional cross-section, with tolerances of 0.5 min, 30 ppm, and 3.0%. Samples and standards were normalized to the abundance of internal standard. Samples were compared with a ‘blank’ consisting of 1X PBS never exposed to cells. Lipids were not quantified if the ion intensity from a given sample was less than the ion intensity of the blank. Lipid concentrations were calculated assuming a 1:1 response ratio as the same-class internal standards. When the same lipid species and tail length was found in two or more adducts, we used the average of molar values for each adduct.

### Trans-epithelial electrical resistance

We measured TEER in RPE plated on transwell filter membranes using an Epithelial Volt-Ohm Meter (Millipore, #MERS00002) and ERS Probes (Millipore, #MERSSTX01). TEER was measured in triplicate for each well and averaged (*TEER*_*well*_). A cell-free TEER which only includes the filter membrane (*TEER*_*blank*_) is also determined and subtracted from *TEER*_*well*_. The resulting TEER (in Ω) is multiplied by the culture surface area *Area*_*membrane*_ to obtain final resistance values (in Ω∗cm^2^). For mixed cellulose ester filters, *Area*_*membrane*_ = 0.6 cm^2^.

### Enzyme-linked immunosorbent assays

For ELISA experiments, cells were treated with vehicle (0.1% ethanol) or indicated concentrations of Firsocostat at 6 weeks of age with medium being changed as indicated above. After 2 weeks of the treatment, the 50 μl of media was collected at 0, 8, 24, and 36 h (Fig. 4*E*), and 24 h post treatment (Fig. 4*F*) for ApoE ELISA. Similarly, the 50 μl of medium was collected after 24 h post treatment for VEGF ELISA.

We used ELISAs to quantify human ApoA1 (R&D biosystems, DY3664), ApoB (R&D biosystems, DAPB00), ApoE (Abcam, ab108813), and VEGF (R&D biosystems, DY293B) content in cell culture supernatant. For each assay, we followed the manufacturer’s instructions, and all quantified data were in the linear range of the standard curve. Absorbance was measured on a BioTek Synergy 4 plate reader.

### Glucose and lactate assays

Glucose and lactate levels in cell culture supernatant were determined enzymatically, as in (74) but adapted for use in a 96-well plate. A detailed description of the adapted glucose assay protocol can be found at https://www.protocols.io/view/glucose-concentration-assay-hexokinase-g6pdh-metho-dm6gpj5jdgzp/v1. A detailed description of the adapted lactate assay can be found at https://www.protocols.io/view/lactate-concentration-assay-ldh-method-6qpvr4733gmk/v1. For each assay, levels of glucose or lactate are determined by measuring absorbance of light at 340 nm, which reflects NADH or NADPH produced in the assay. We measured absorption of 340 nm light using a Synergy 4 plate reader (BioTek).

### Cholesterol assays

We measured the content of total (esterified and unesterified) cholesterol in 50 μl of cell culture medium supernatant using a commercial enzymatic assay (Caymen chemical, 10007640), following the manufacturer’s protocol and reading fluorescence at an excitation wavelength of 535 nm and an emission wavelength of 590 nm. One exception was made to the manufacturers protocol; in addition to reading fluorescent emission at 30 min, we read the emission over the course of 1 h at 37 °C after combining the sample and reaction mixture, to confirm that the peroxidase reaction had completed.

### Lactate dehydrogenase assays

We quantified LDH levels using a CyQuant Cytotoxicity Assay kit (Thermo Fisher Scientific, C20300) and followed the manufacturers protocol. Positive LDH controls were fully lysed in 10% Triton-X100; negative controls were cell-free culture medium. We confirmed that all absorbance measurements were in the linear range of the reaction using a BioTek Synergy 4 plate reader.

### Sectioning and Immunofluorescent labeling

For immunohistochemical analysis of protein localization, cells were fixed using 4% paraformaldehyde in 1x PBS (pH 7.4) for 24 h. Filters were then washed in PBS and quartered. Filter quarters were embedded in OCT then sectioned at −20 °C at a 20 μm thickness. Sections were stored at −80 °C.

Filter sections were warmed for 30 min at 50 °C to adhere filters to slides, and an oil pen was used to form a hydrophobic barrier on slides. Slides were washed in 1× PBS, pH 7.4, nonspecific antigens blocked using 2% donkey serum in PBS, incubated in primary cocktail made of goat α-ApoE (EMD-Millipore, AB947, 1:500), Hoechst-33258 (Thermo Fisher Scientific, H3570, 5 μg/ml), and phalloidin-568 (Molecular Probes, A12380, 2U/ml) overnight at 4 °C, washed three times, and labeled with secondary antibody (AlexaFluor633-conjugated donkey α-goat, Invitrogen, A21082, 1:1000) for 2 h at room temperature.

For imaging of lipid droplets, membranes were washed, blocked without a permeabilization step, then stained *en face* with Bodipy-493/503 (Thermo Fisher Scientific, D3922, 12.6 μM, Hoechst-33258, and phalloidin-568), overnight at 4 °C.

Fluorescent sections and *en face* filters were washed twice in 1× PBS and mounted in fluoromount G and sealed with clear nail polish. Slides were imaged on a Leica SP8 confocal microscope. Each image covered a 144.87 μm (x) by 144.87 μm (y) area (at 1024 x 1024 pixels). For each imaging area, eleven z-stack images were taken covering a 10 μm thickness. Nine to ten replicate images were taken from different regions of each RPE mixed cellulose ester membrane.

### Matrix metalloproteinase activity assay

We determined matrix metalloproteinase activity using an assay (Abcam, ab112147) containing a FRET peptide with self-quenching fluorescence. Cleavage of this peptide by matrix metalloproteinases results in a fluorescent signal at 590 nm when excited at 540 nm. The assay began by incubating RPE culture medium samples (50 μl/sample) with 50 μl of 2 mM 4-aminophenylmercuric acetate, for 90 min at room temperature. Fifty microliters of the FRET peptide was then added and fluorescence tracked for 30 min. The rate of change in fluorescence over time was determined, and the rate of change of the ‘background’ sample consisting of medium that had not touched cells was subtracted from all samples. The resulting rates of substrate cleavage are referred to as ‘arbitrary units’.

### Image analysis

ApoE puncta are quantified in Figures 6*D* and 7*G*. Image processing and puncta quantification used ImageJ (v1.53t). Image stacks were made into a maximum intensity projection prior to downstream analysis. The ApoE channel of this projection was processed with a gaussian blur function (sigma = 1). Background was subtracted using a sliding paraboloid function, then an intensity threshold was applied (30–255). The number, size, and coverage of the ApoE channel were determined using the “analyze particles” tool. A flow chart of the analysis performed on a sample image is available as Fig. S1. Images displayed in Figure 6, *A*–*C* are background subtracted. Particle analysis results were averaged, such that a single point represents the average of 9 to 10 images made across that membrane. Different replicates represent different membranes.

### Statistical analysis

All displayed data include mean ± standard error and may additionally show individual experimental replicates. Statistical analyses were performed using Prism v10.0 (GraphPad Software). Significance thresholds were set to α < 0.05. When below this threshold, statistical values are noted within the figure. Statistical tests are as follows: Two-tailed Student’s *t* test: Figures 1*C*, and 3*C*, *G*–*I*; 1-way ANOVA: Figures 1, *E*–*H*, 2*G*, 4, *D*–*H*, 5*D*, and 6, *F*, and *H*; 2-way ANOVA: Figures 1, *I* and *J*, 2*H*, 3*B*, 5, *G*, and *J*, and 6, *E* and *K*. All *post hoc* hypotheses were tested using the two-stage linear step-up procedure of Benjamini, Krieger, and Yekutieli. Where *p*-values are not directly indicated, ∗*p* < 0.05, ∗∗*p* < 0.01, ∗∗∗*p* < 0.001.

## Data availability

Data are available upon request. Requests should be submitted to Daniel Hass at dhass@uw.edu.

## Supporting information

This article contains supporting information.

## Conflicts of interest

The authors declare that they have no conflicts of interest with the contents of this article.

## Figures and Tables

**Figure S5:**